# Transfer of Training from Virtual to Real Baseball Batting

**DOI:** 10.3389/fpsyg.2017.02183

**Published:** 2017-12-13

**Authors:** Rob Gray

**Affiliations:** Human Systems Engineering, Arizona State University Polytechnic Campus, Mesa, AZ, United States

**Keywords:** virtual environments, transfer of training, baseball, perception-action, practice design

## Abstract

The use of virtual environments (VE) for training perceptual-motors skills in sports continues to be a rapidly growing area. However, there is a dearth of research that has examined whether training in sports simulation transfers to the real task. In this study, the transfer of perceptual-motor skills trained in an adaptive baseball batting VE to real baseball performance was investigated. Eighty participants were assigned equally to groups undertaking adaptive hitting training in the VE, extra sessions of batting practice in the VE, extra sessions of real batting practice, and a control condition involving no additional training to the players’ regular practice. Training involved two 45 min sessions per week for 6 weeks. Performance on a batting test in the VE, in an on-field test of batting, and on a pitch recognition test was measured pre- and post-training. League batting statistics in the season following training and the highest level of competition reached in the following 5 years were also analyzed. For the majority of performance measures, the adaptive VE training group showed a significantly greater improvement from pre-post training as compared to the other groups. In addition, players in this group had superior batting statistics in league play and reached higher levels of competition. Training in a VE can be used to improve real, on-field performance especially when designers take advantage of simulation to provide training methods (e.g., adaptive training) that do not simply recreate the real training situation.

## Introduction

In recent years there has been a renewed interest in using virtual environments (VEs)^1^ as a tool for training perceptual–cognitive skills in sports (Miles et al., 2012). This has been motivated by two potential benefits that have been identified. First, using a sports VE creates the opportunity to train under conditions that are impossible in the real world. For example, attempting to catch a virtual ball that does not obey the laws of gravity (Zaal and Bootsma, 2011) or one for which there is a conflict between perceptual information sources (Gray and Sieffert, 2005). Second, using a sports VE allows some of the key evidence-based principles of practice design (Hendry et al., 2015) to be more easily and effectively incorporated into training. These include adding a high degree of variability to practice conditions and systematically adjusting the level of challenge based on the athlete’s performance. The goal of the present study was to evaluate the effectiveness of a type of VE training designed to take advantage of potential practice design benefits.

As discussed in Gray (in press), the primary criterion for any training VE should always be positive transfer of training to the real environment. Transfer of training can be defined as the gain (or loss) in the capability for performance of one task as a result of practice on some other task (Schmidt and Lee, 1982). Secondary criteria for VE evaluation (including fidelity, immersion, and technical specifications such as the size of the field of view) should only be considered important to the extent to which they aid in creating positive transfer of training. While it has been shown that sports VEs have many of the characteristics necessary (but not sufficient) for effective training outcomes (reviewed in Gray, in press), there have been relatively few studies that have directly assessed transfer for VEs designed for sports training.

In evaluating transfer from any sports training there are several important research design factors that must be taken into account (Abernethy and Wood, 2001). First, does the study include appropriate controls to rule out placebo and basic practice effects? Second, does the study include some assessment of the underlying mechanism(s) the training is purported to improve? For example, if a new training program is designed to improve sports performance by improving the clarity of vision, is visual acuity assessed pre- and post-training using a test that is validated and is preferably different than the task used during training? This helps to assess to what extent any improvements in sports performance are directly related to the training. Finally, does the study include some assessment of far transfer? “Far” in this context refers to the distance between the task performed in training (the transfer task) and the actual sport (the criterion task) (Abernethy and Wood, 2001). Solely assessing near transfer of training (e.g., quantifying to what extent athletes improve on the task used during training) is insufficient for assessing the value of a sports VE because near transfer is almost always positive and large in magnitude while producing positive, far transfer is much more difficult (Schmidt and Young, 1987). Therefore, it is critical that transfer studies include some measurement of far transfer involving a task that is close to what is performed during actual competition. The small number of transfer studies that have been published are examined next using these criteria.

Todorov et al. (1997) investigated transfer of training for a virtual table tennis trainer. In this study, a group that was trained by a coach was compared to a group training in a VE which contained an image of the table, ball, and the user’s paddle (which was linked to their actual paddle movements using a motion tracker). A second paddle was also displayed in the simulation which showed the movements of an expert player (the coach) when hitting a shot. In a first experiment, a control group that received traditional coaching was compared to a VE training group. Pre- and post-training tests involved hitting a ball at a target on a real table. In terms of target hits, the group that trained in the VE showed a significantly greater improvement than the traditional training group.

In a second experiment, a more difficult shot task was used in which a barrier was placed on the table that participants could either attempt to hit over or under. Again, the increase in the number of targets hit from pre–post-training was significantly greater for the group that trained in the VE as compared to a traditional training group. An analysis of stroke kinematics revealed the differences between the paddle movement between each participant and the expert coach were significantly smaller for players in the VE training group. However, for the VE group, this difference increased throughout training and segments of the stroke (e.g., the backswing) were often completely different from the pattern of the coach by the end of training. This suggests participants were not simply copying the movements of the coach’s racquet in the VE but were rather using them as a guide to find their own individual perceptual-motor solution to the task. Overall, this study includes most elements of a good transfer study and provides evidence of the potential value of VE training in sports. The only element that is missing is a measure of far transfer in which performance during a table tennis match is assessed in some way.

Lammfromm and Gopher (2011) examined transfer for a juggling VE. The VE was a very low fidelity simulation in which participants controlled simulated representations of their hands to juggle balls in a 2D wall display. So essentially the simulation could be used to learn some of the perceptual–cognitive aspects of juggling, but did not accurately recreate the motor component. The VE had the advantage that it allowed participants to practice juggling at lower speeds than are possible in real juggling and gradually increase speed as they improve. A group that performed both real and virtual juggling training was compared with a control group that was trained in real juggling alone. In terms of the number of consecutive juggling cycles that could be performed, both training groups improved by the same amount pre–post-training when tested at typical juggling speeds. However, when forced to juggle at higher speeds, the group that received the additional VE training performed significantly better than the real training only group. While this study again provides some evidence for the benefits of VE training, it would have been useful to include a VE training only group and some assessment of the performance changes (e.g., kinematics, eye movements) to determine exactly what value VE training was adding.

Rauter et al. (2013) investigated transfer of training for a very high fidelity rowing simulation. This VE included a large CAVE display of the water, realistic sounds of the boat moving through the water, and oars that were attached to a series of ropes that delivered highly realistic haptic feedback to the rower. In the training study, four participants trained in the simulation and four did real training on the water where they received verbal feedback from a coach. Pre- and post-training tests (which involved participants attempting to produce their best rowing technique) were conducted in both the simulator and on water. Performance was evaluated primarily using biomechanical measures specific to rowing technique. The results provided evidence for transfer in both directions. That is, participants that trained in the VE showed significant improvement (pre–post-training) for several of the biomechanical measures when tested on open water while participants trained on open water show significant improvements when tested in the VE. Although this pilot study provides interesting results, it is limited by its low sample size and lack of a measure of far transfer (e.g., time to complete a rowing race).

Finally, Tirp et al. (2015) examined transfer of training from the darts game in the Microsoft Kinect VE to real darts. In this study three groups were compared: a group that practiced real darts, one that practiced in the VE, and a control group that did not practice. Pre- and post-tests involved executing 15 shots at the bullseye on a real dartboard. A unique aspect of this study was that the quiet eye duration (i.e., the amount of time the thrower fixated on the target before releasing the dart, Vickers, 1992) was measured. Both the real and virtual training groups showed improvements (from pre–post-training) in throwing accuracy that were significantly greater than for the control group. However, performance improvements were significantly greater for the real training group than the VE training group. The quiet eye duration increased significantly for both groups after training with the increase being significantly larger for the VE group. In sum, this study provides somewhat equivocal results with regards to the benefits of VE training. While the gaze behavior change seems to indicate the VE training was comparable or even superior to real training, this was not borne out in actual throwing performance.

From this review of existing research on the topic, it is abundantly clear that more work is needed to determine whether training in a sports VE will produce positive transfer of training to real sport. Without effective evaluation of transfer it will continue to be difficult for sports teams to determine whether a VE is worth the investment and to determine which technological components are required for training success. While existing studies are generally well designed they are small in number and none involve tests of far transfer (i.e., performance in actual competition or competition-like conditions). Furthermore, from existing research it is unclear whether a sports training VE’s value will come from just giving an athlete more repetitions or “reps” of the skill (i.e., recreating real training) or whether it will come from taking advantage of VEs to design types of practice that are difficult or impossible to do in real life (as suggested by the table tennis and juggling results described above) or both.

The goal of the present study was to address these limitations by examining the transfer of perceptual-motor skills trained in an adaptive baseball batting VE to real baseball performance. Eighty participants who were taking part in regular training were assigned equally to groups undertaking adaptive hitting training in the batting VE, extra sessions of batting practice in the VE, extra sessions of real batting practice, and a control condition involving no additional training to the players’ regular practice. The adaptive training involved performance-based adjustments of pitch speed, pitch type, and location using staircase methods. The batting practice training (both real and VE) involved blocked practice of different pitch types with speed and location held constant, as is typical in baseball (e.g., American Baseball Coaches Association, 2009). Performance on a batting test in the VE, in an on-field test of batting, and on a pitch recognition test was measured pre- and post-training. The league batting statistics for the season following training and the highest level of competition reached in a 5-year period following the training were also analyzed. The experiment was designed to test the following specific hypotheses:

(1) For all performance measures, the change from pre–post-training would be significantly greater for the VE adaptive training group than for all other groups. This was predicted because (as described in detail below) the adaptive training involved taking advantage of the VE to incorporate evidence-based training elements that are not typically used in real training.
(2) Batting performance in the season following training would be significantly greater for the VE adaptive training group than for all other groups.
(3) A significantly greater proportion of batters in the VE adaptive training group would reach a level of competition higher than high school baseball than for all other groups.

## Materials and Methods

### Participants

The participants in the study were 80 male baseball players who played competitive high school baseball in the United States at the time of training. The sample size of 20 per group was determined based on power analysis (power = 0.8) using the mean effect size (*f* = 0.75) from previous studies comparing batters of different skill levels using the same batting VE (Gray, 2002, 2004; Castaneda and Gray, 2007) with the goal of having sufficient power to detect group × phase (i.e., pre–post-training) interactions. All players were in their senior year and were either 17 or 18 years of age. Players were recruited, trained, and tested over a 3-year period from 2008 to 2010. They were recruited from 18 different teams and all players started the majority of the games at their position the previous season. This study was carried out in accordance with the recommendations of and was approved by the Arizona State University Institutional Review Board with written informed consent from all subjects. All subjects gave written informed consent in accordance with the Declaration of Helsinki.

The 80 participants were randomly assigned to one of four training groups (described in detail below): (i) Adaptive training in a batting VE, (ii) extra sessions of batting practice in the VE, (iii) extra on-field sessions of real batting practice, and (iv) a control condition involving no additional training to the players’ regular practice. The mean number of years of competitive playing experience for the four groups was, respectively, 8.6 (*SD* = 1.2), 8.3 (*SD* = 0.9), 8.8 (*SD* = 1.4), and 8.5 (*SD* = 1.1). A one-way ANOVA revealed that there was no significant difference in the number of year of competitive playing experience, *p*> 0.5, $\eta_{p}^{2}$ < 0.1.

### Apparatus

The baseball batting VE used in the present study has been used in several previous experiments (e.g., Gray, 2002, 2004, 2009a,b; Castaneda and Gray, 2007). Briefly, participants swung a baseball bat at a simulated approaching baseball. The image of the ball, a pitcher, and the playing field was projected on a single 6.9′ (2.11 m) (h) × 4.8′ (1.47 m) (v) screen positioned in front of the batter using a Proxima 6850+ LCD projector updated at a rate of 60 Hz. The flight of the ball was simulated until it was approximately 5 feet (1.7 m) from the front of the plate so batters could not see the virtual ball as it crossed the plate. The bat was not simulated in the visual display so participants could not see the point of bat–ball contact. Research on gaze behavior in baseball suggests that the ball will be well outside foveal vision at a distance of 5 feet for most batters (Bahill and LaRitz, 1984). The importance of the fidelity of the VE used in the present study is considered below.

Mounted on the end of the bat [Rawlings Big Stick Professional Model; 33″ (84 cm)] was a sensor from a Fastrak (Polhemus) position tracker. The sensor was not wireless so the position of the cord was adjusted after each trial so as not to interfere with the batter’s swing. All of the batters in the study reported that they could swing freely and naturally. The *x, y, z* position of the end of the bat was recorded at a rate of 120 Hz. The position of the ball in the simulation was compared with the recording of bat position in real-time in order to detect collisions between the bat and ball. Batters received visual, auditory, and tactile feedback about the success of their swing [see Gray (2009b) for details]. Three pitch types were used: (i) a “four seam” fastball with an average speed of 85 mph (38 m/s), thrown with backspin, and with a spin rate of 1900 rpm, (ii) a “12–6” curveball with an average speed of 65 mph (29.0 m/s), thrown with topspin, and with a spin rates of 1700 rpm, and (iii) a “straight change” with an average speed of 70 mph (31.2 m/s), thrown with backspin, and with a spin rate of 1800 rpm. As described in detail below, both right-handed and left-handed pitchers were simulated with the ratio of their usage in training and testing (75% right-handed) roughly reflecting the typical ratio found in baseball.

For real batting practice training and on-field tests, batters hit balls projected by a Rawlings Spin Ball Pro 3 Spin Wheel^TM^ pitching machine. The same three pitch types were used and the pitching machine was moved to different sides of the pitching rubber to simulate left- and right-handed deliveries.

### Procedure

#### Pre-tests

Prior to training, all batters completed three pre-test performance assessments: VE batting, real batting, and pitch recognition in the VE. For practical reasons, the two VE tests were always performed in the same session while the real batting test was performed in a separate session. The order of the VE and real tests was fully counterbalanced across participants (*n* = 10 per order) as was the order of the hitting and pitch recognition tests within the VE session (*n* = 5 per order). The details of the three tests were as follows:

##### VE batting test

In this test, batters faced a series of pitches until the sum of the number of strikes plus the number of hits was equal to 20. A strike occurred when the batter swung and missed the ball, the batter did not swing at a ball that crossed the plate in the strike zone, the batter hit a ball that did not make it to the outfield, or the batter hit the ball into foul territory. Hits included homeruns (balls hit further than 320 feet) and balls that landed in fair play beyond the infield. If the batter did not swing and the ball crossed the plate outside the strike zone, the pitch was not added to their total, i.e., the batter could “take” pitches. All definitions of a “hit” and how their performance would be scored was explained to each batter before they were tested. The motion tracker was used to determine whether or not the bat crossed the front of the plate for swinging strike calls.

The lateral location and height of each pitch when it crossed the plate was varied to simulate pitches that were “strikes” (i.e., crossed the plate in the strike zone) and pitches that were “balls” (i.e., did not cross in the strike zone). The Major League Baseball (MLB) definition of the strike zone (Triumph Books, 2004) was used to determine balls and strikes: “the strike zone is that area over home plate the upper limit of which is a horizontal line at the midpoint between the top of the shoulders and the top of the uniform pants, and the lower level is a line at the top of the knees.” “Strikes” and “balls” were selected randomly for each pitch with a “strike” probability of 0.65. Pitch type (fastball, curveball, or changeup) was also chosen randomly on each trial. For each pitch type, there were 10 different combinations of pitch parameters (horizontal and vertical launch angle and speed) that resulted in strikes and 9 different combinations of pitch parameters that resulted in balls. The range of pitch speeds was ±5 mph (2.2 m/s) around the average speed for each pitch type described above. Strikes were spread equally throughout the strike zone while pitches that were not strikes crossed the plate either above or below the strike zone or were outside (i.e., on the side of the plate opposite to where the batter was standing). All balls missed the strike zone by 4″ (10 cm). Pitches that were off the plate inside were not used because this condition was not included in the real batting test for safety reasons.

The simulated pitcher was right-handed for the first 15 strikes + hits then was switched to left-handed for the remainder of the test. Batters hit from their preferred side of the plate and were allowed to switch-hit (i.e., switch sides when the simulated pitcher handedness was changed) if they wished. Batters were given 10 min breaks after every 20 pitches to reduce fatigue. Batters were told the definition of hit, how many pitches they would receive, that taking pitches outside the strike zone would not count to their total, and to “try and get as many hits as possible.”

##### Real batting test

In this test, batters attempted to hit regulation baseballs thrown by a pitching machine. The procedure was identical to that described for the VE test except for balls and strikes were called by umpires with a minimum 5 years of experience. Pitching machine settings for the different pitch outcomes were determined in pilot testing.

##### Pitch recognition test

In this test, batters passively viewed pitches in the batting VE that were occluded (and replaced with a blank screen) 150 ms after release. This viewing duration was chosen based on previous research suggesting that this is roughly the point at which a batter must make a decision about whether or not to complete a full swing (Gray, 2009a). There were a total of 20 pitches in the test. The pitch parameters were identical to that described for the VE batting test. Batters were given the following instruction: “for each pitch your task is judge the pitch type (fastball, curveball, or changeup) and whether the pitch was a strike or a ball as accurately and as quickly as possible. You should make your response verbally and indicate the pitch type first.” The experimenter recorded the responses for each pitch and response time was not calculated. Batters were not given feedback about the accuracy of their judgments.

#### Training

All three training groups completed two 45 min sessions per week for 6 weeks. All training was completed in the year prior to players’ final season of high school baseball. Details of the training sessions were as follows:

##### Batting practice in the VE

In each session, batters attempted to hit 30 pitches with the instructed goal of “attempting to hit the ball hard over the infield.” All pitches were strikes and traveled down the center of the strike zone. The three pitch types were blocked with 10 pitches per type and the order randomized in each session. The initial pitch speeds for each type were 80 mph (38 m/s) fastball, 65 mph (29 m/s) curveball, and 70 mph (31.2 m/s) changeup. In each session, the pitcher had a constant handedness with the first nine training sessions using a right-handed pitcher and the final three using a left-hander. After every three sessions, the speed of each pitch was increased by 1 mph (0.45 m/s). The design of this training was based on what is typically done in real baseball. For example, pitch type and pitcher handedness were blocked and speeds were not varied within pitch type because it is impractical to vary these parameters from pitch to pitch in a real training session.

##### Real batting practice

This training was identical to the VE batting practice group except that, of course, batters attempted to hit real balls thrown by a pitching machine.

##### Adaptive VE training

As was the case in the other two training groups, batters attempted to hit 30 pitches per training session with the first nine training sessions using a right-handed pitcher and the final three using a left-hander. However, the design of this training was based on previous research demonstrating that training outcomes are improved when practice is designed so that the task difficulty is appropriately matched to the performer’s skill level (i.e., the challenge point hypothesis, Guadagnoli and Lee, 2004) and includes variability in practice conditions (Schmidt, 1975).

**FIGURE 1:**
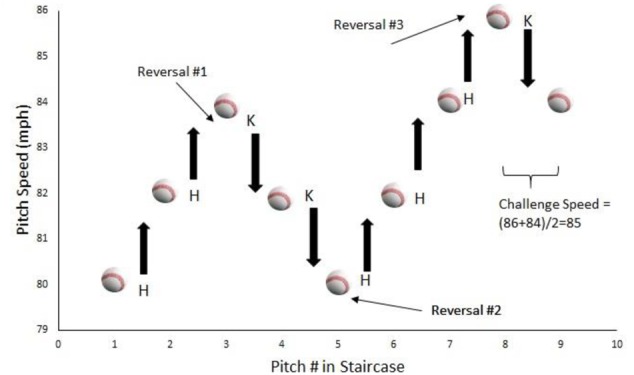
Example of the staircase procedure used for the adaptive VE training group. After each successful hit (H) the pitch speed was increased by 2 mph while after each strike (K) it was decreased by the same amount. After three reversals, the challenge speed was determined by averaging the pitch speed on the final two trials.

To manipulate challenge, the pitch parameters in the batting VE were determined by three one-up-one-down staircases (Levitt, 1971), with one staircase corresponding to each pitch type. An example staircase for the fastball is shown in **Figure 1**. At the start of training, the pitch speed (i.e., the initial value in the staircase) was the mean for that particular pitch type. All pitches were initially strikes that traveled down the center of the strike zone. If the batter successfully achieved a hit for this pitch, the speed was increased by 2 mph (0.9 m/s) for the next pitch in that staircase. If the result of the pitch was a strike (denoted by ‘K’ in **Figure 1**), the speed was decreased by 2 mph (0.9 m/s). After three reversals (i.e., trails for which the outcome was opposite to what occurred on the previous trial), the “challenge speed” was determined by calculating average speed for the last two trials of that staircase. The pitch speed was then held constant at this value. Another way of thinking of this manipulation is the following. The simulation program altered the pitch speed until a “threshold” value was found for which an increase in speed would typically cause the batter to not get a hit while a decrease would lead to hits for most pitches. See Gray and Allsop (2013) for a similar procedure.

After this challenge speed value was found, the variability in the pitch crossing height was next varied. Specifically, instead of always traveling down the center of the strike zone, pitch crossing height varied randomly between ±*y* cm around the center. The initial value of *y* was 2″ (5 cm). The value of *y* was increased by 2″ (5 cm) after each hit and was decreased by 2″ (5 cm) after each strike. After three reversals or if the batter achieved three consecutive hits for the maximum *y* value (9.8″, 25 cm), the “challenge crossing height variability” was set. The final manipulation involved an analogous adjustment of the variability of the lateral crossing location.

At the start of each new training session, the initial pitch parameters were equal to the final settings from the previous session. Once the three challenge points (speed, crossing height, and lateral location) were determined these values were used for the next full training session. After this, the entire procedure started over (i.e., a new speed challenge point was determined, etc.). The entire procedure was also started over for the final three sessions in which the batter faced the left-hander pitcher. To manipulate the variability of practice conditions the three staircases (each corresponding to one pitch type) were randomly interleaved during each session.

#### Post-tests

Post-tests were identical to the pre-tests and were conducted roughly 2 weeks after the final training session for all participants.

#### Retention Tests

Retention tests were identical to the pre- and post-tests and were completed roughly 1 month after the post-test. The retention tests were included to determine to what extent any training benefits were maintained after training ended.

### Data Analysis

#### Batting Performance Assessments

For the VE and on-field batting tests the following dependent variables were analyzed: total number of hits, % of swings at pitches inside the strike zone (Z-Swing %), and % of swings at pitches outside of the strike zone (O-Swing %).^2^ These variables were chosen because they reflect both a player’s hitting ability and their knowledge of the strike zone. For the pitch recognition test, the total number of pitch types and balls/strikes correctly identified were used as dependent variables. These variables were first analyzed using a 3 (testing phase: pre, post, retention) × 4 (group: adaptive VE, VE batting practice, real batting practice, control) MANOVA with significant effects further analyzed using ANOVAs and *t*-tests.

#### Five-Year Follow Up

For all participants, we calculated the on-base percentage (OBP) for their senior high school season following the training. OBP is a measure of how often a player reaches base with the exact formula, OBP = Hits + Walks + Hit by Pitch)/(At Bats + Walks + Hit by Pitch + Sacrifice Flies). This variable was chosen because it captures both a player’s ability to hit and their knowledge of the strike zone, both of which were targeted in training. OBP data for the four training groups were first analyzed using a one-way ANOVA. Next, we sought to determine which of the dependent variables in the batting assessments were significantly related to OBP. To achieve this end, a linear multiple regression was performed with OBP as the dependent variable and change scores (from pre- to post-training) for batting assessments as independent variables.

For the four training groups, the highest level of competition for which each player competed at least one full season within the 5 years following training was determined. This included NCJAA junior college, NCAA college, or any level of MLB (e.g., A, AA, AAA). The proportion of players reaching a level above high school baseball was compared for the groups using a Chi-square test of proportions.

## Results

### Batting Performance Assessments

**FIGURE 2:**
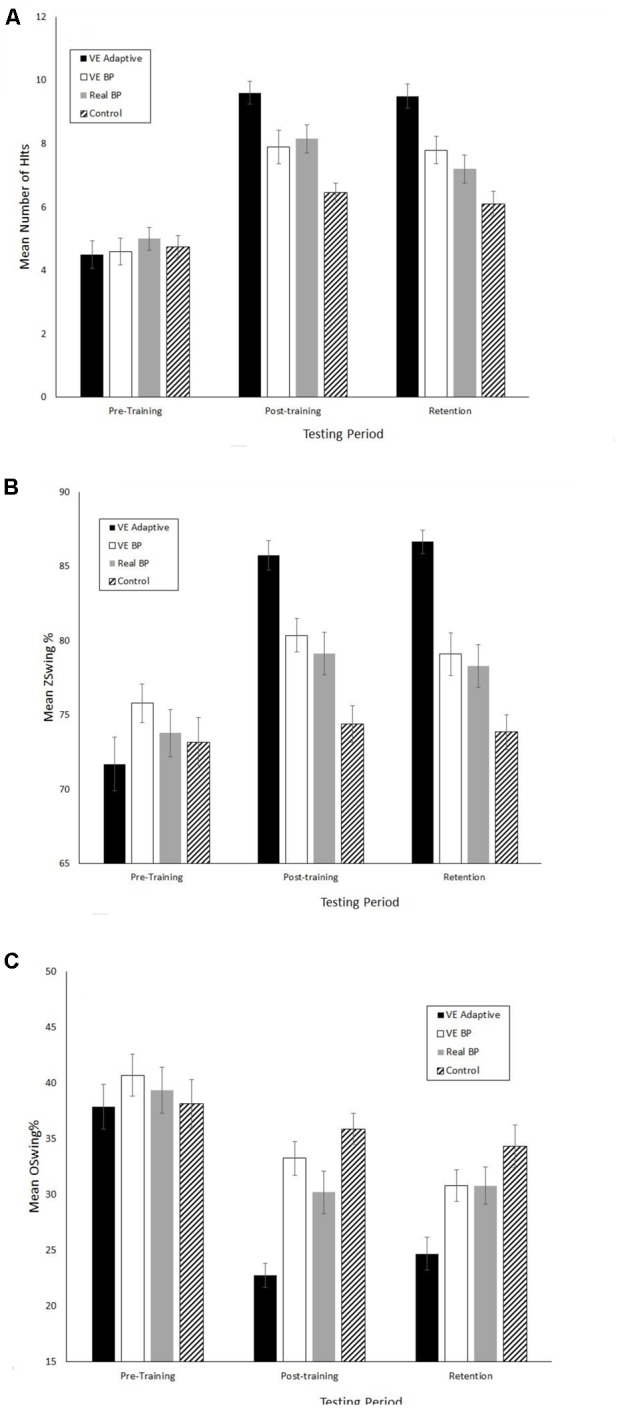
Mean performance scores for the VE batting tests. **(A)** Number of hits. **(B)** Z-Swing %. **(C)** O-Swing %. Error bars are standard errors.

**FIGURE 3:**
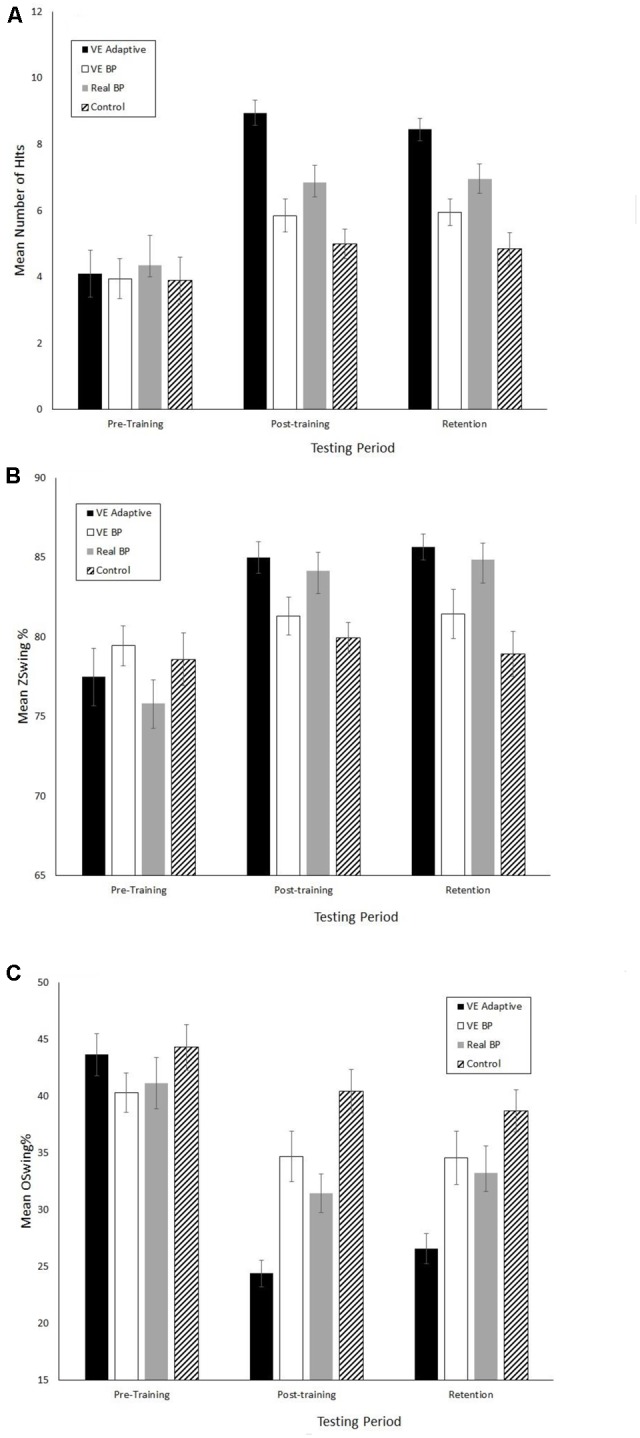
Mean performance scores for the real batting tests. **(A)** Number of hits. **(B)** Z-Swing %. **(C)** O-Swing %. Error bars are standard errors.

**FIGURE 4:**
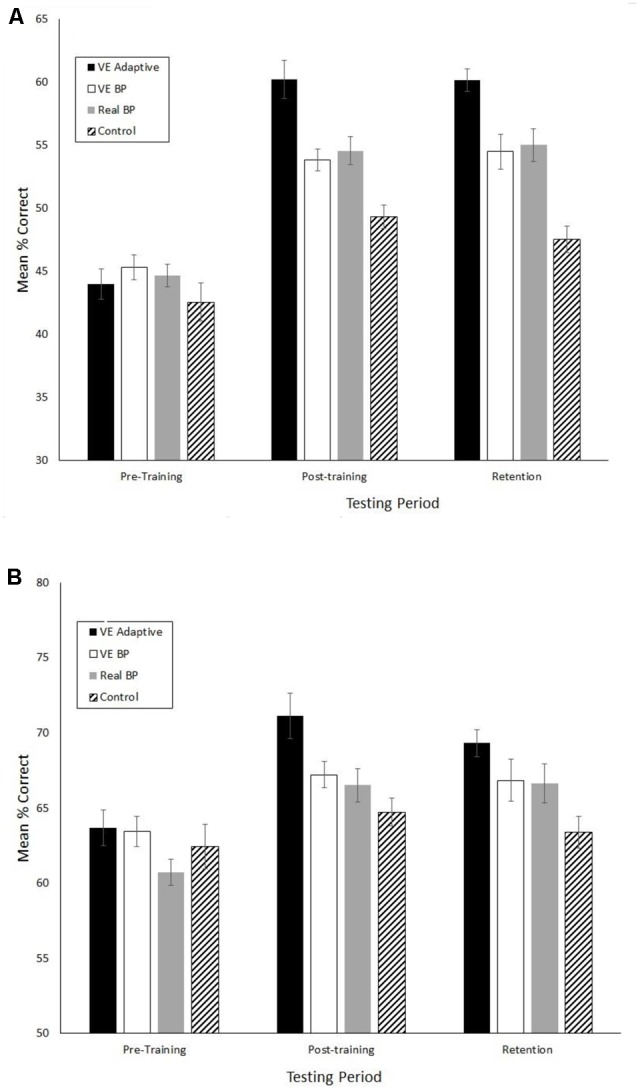
Mean accuracy for the pitch type **(A)** and strike/ball **(B)** judgments in the pitch recognition test. Error bars are standard errors.

**Figures 2–4** show the mean values for the performance assessment variables in three phases of the study, respectively. The MANOVA performed on the eight batting assessment variables revealed significant main effects of group, *F*(8,69) = 4.3, Wilks *λ* = 0.17, *p*< 0.001, $\eta_{p}^{2}$ = 0.33, phase, *F*(16,61) = 49.3, Wilks *λ* = 0.07, *p*< 0.001, $\eta_{p}^{2}$ = 0.93, and a significant group × phase interaction, *F*(48,189) = 2.0, Wilks *λ* = 0.19, *p* = 0.001, $\eta_{p}^{2}$ = 0.34.

The results of the 3 × 4 ANOVAs performed on each of the dependent variables are shown in **Table 1**. For all variables there were significant main effects of group and phase. With the exception of the total number of balls/strikes correctly identified, these effects were qualified by significant group × phase interactions for all dependent variables. In the following sections, these effects are further broken down by the phases of the experiment.

**Table 1:** Results of the 3 × 4 ANOVAS performed on the Batting Assessment Variables.

Dependent variable	Group *F*(3,76)	Phase *F*(2,152)	Group × Phase *F*(6,152)
VE_Hits	12.3*^∗^*, $\eta_{p}^{2}$ = 0.33	82.6*^∗^*, $\eta_{p}^{2}$ = 0.52	4.8*^∗^*, $\eta_{p}^{2}$ = 0.16
VE_ZSwing	13.3*^∗^*, $\eta_{p}^{2}$ = 0.34	29.6*^∗^*, $\eta_{p}^{2}$ = 0.28	7.0*^∗^*, $\eta_{p}^{2}$ = 0.22
VE_OSwing	10.0*^∗^*, $\eta_{p}^{2}$ = 0.29	35.5*^∗^*, $\eta_{p}^{2}$ = 0.32	2.8^b^, $\eta_{p}^{2}$ = 0.10
Real_Hits	15.1*^∗^*, $\eta_{p}^{2}$ = 0.37	51.5*^∗^*, $\eta_{p}^{2}$ = 0.40	4.7*^∗^*, $\eta_{p}^{2}$ = 0.16
Real_ZSwing	2.9^a^, $\eta_{p}^{2}$ = 0.11	21.5*^∗^*, $\eta_{p}^{2}$ = 0.22	3.8*^∗^*, $\eta_{p}^{2}$ = 0.13
Real_OSwing	11.4^∗^, $\eta_{p}^{2}$ = 0.31	37.2*^∗^*, $\eta_{p}^{2}$ = 0.33	4.3*^∗^*, $\eta_{p}^{2}$ = 0.14
PR_Type	40.7^∗^, $\eta_{p}^{2}$ = 0.62	152.0*^∗^*, $\eta_{p}^{2}$ = 0.67	7.2*^∗^*, $\eta_{p}^{2}$ = 0.22
PR_Strike	8.5^∗^, $\eta_{p}^{2}$ = 0.25	21.6*^∗^*, $\eta_{p}^{2}$ = 0.22	1.5^c^, $\eta_{p}^{2}$ = 0.06

*^∗^p< 0.001, ^a^*p* = 0.037, ^b^*p* = 0.013, ^c^*p* = 0.18.*

#### Pre-test

To determine whether there were any pre-test group differences for any of the dependent variables, scores were compared using independent samples *t*-tests with Bonferroni correction (critical *p* = 0.008). These analyses revealed no significant group differences for any of the dependent variables, *p*s all > 0.05, *d*s all < 0.5.

#### Post-test

To break down the significant group × phase interactions, pre- and post-test scores were compared separately for each of the training groups using pairwise *t*-tests with Bonferroni correction (critical *p* = 0.006). The results of these analyses are shown in **Table 2**. The VE adaptive training group had significant pre–post improvements for all dependent variables. For the real batting practice group, there were significant improvements for 7/8 of dependent variables, with no significant effect for Z-Swing % in the VE batting test. For the VE batting practice group, there were significant improvements for 3/8 of the variables (number of hits in the VE batting test, O-Swing% in the VE batting test, and number of correct pitch identified in the recognition test). Finally, for the control group, there were significant improvements for 2/8 of the variables (number of hits in the VE test and number of pitch types correctly identified in the recognition test).

**Table 2:** Results of pairwise *t*-tests comparing pre- and post-test scores.

Group	Dependent variable	*t*(19)	*p*	*d*
VE_Adaptive	VE_Hits	9.4*	<0.001	2.8
	VE_ZSwing	7.5*	<0.001	2.1
	VE_OSwing	–7.1*	<0.001	1.9
	Real_Hits	13.9*	<0.001	1.9
	Real_ZSwing	8.5*	<0.001	1.2
	Real_OSwing	–10.6*	<0.001	2.8
	PR_Type	9.2*	<0.001	2.7
	PR_Strike	6.0*	<0.001	1.4
VE_BP	VE_Hits	4.8*	<0.001	1.5
	VE_ZSwing	2.4	0.027	0.6
	VE_OSwing	–3.2*	0.005	0.9
	Real_Hits	2.5	0.018	0.8
	Real_ZSwing	1.2	0.23	0.3
	Real_OSwing	–1.7	0.10	0.6
	PR_Type	7.0*	<0.001	2.1
	PR_Strike	2.8	0.01	0.7
Real_BP	VE_Hits	6.0*	<0.001	1.7
	VE_ZSwing	2.6	0.018	0.7
	VE_OSwing	–3.4*	0.003	1.0
	Real_Hits	4.3*	<0.001	0.8
	Real_ZSwing	5.1*	<0.001	1.4
	Real_OSwing	–3.3*	0.004	1.1
	PR_Type	7.5*	<0.001	2.2
	PR_Strike	3.5*	0.003	1.1
Control	VE_Hits	3.9*	<0.001	1.1
	VE_ZSwing	0.83	0.42	0.2
	VE_OSwing	–1.09	0.29	0.3
	Real_Hits	2.2	0.043	0.4
	Real_ZSwing	0.66	0.52	0.2
	Real_OSwing	–1.8	0.087	0.4
	PR_Type	5.0*	<0.001	1.2
	PR_Strike	1.6	0.13	0.5

*^∗^Significant at corrected *p* = 0.006.*

To test hypotheses (i), pre- to post-test change scores were calculated for each group and each dependent variable. The change score for the VE adaptive group was compared to each of the other groups using independent samples *t*-tests with Bonferroni correction (critical *p* value = 0.006). The results of these analyses are shown in **Table 3**. In comparison to the VE batting practice group, the VE adaptive training group had significantly greater change scores for 5/8 of the dependent measures (Z-Swing % in the VE batting test; number of hits, Z-Swing % and O-Swing% in the real batting tests; number of correct pitch types identified). In comparison to the real batting practice group, the VE adaptive training group had significantly greater change scores for 5/8 of the dependent measures (number of hits in the VE batting test; number of hits and Z-Swing % in the real batting tests; number of correct pitch types identified). Finally, as compared to the control group, the VE adaptive training group had significantly greater change scores for 7/8 of the dependent measures with the only non-significant difference occurring for the number of strikes correctly identified.

**Table 3:** Results of *t*-tests comparing the VE adaptive group to other training groups.

Dependent variable	Group	*t*(38)	*p*	*d*
VE_Hits	VE_BP	2.05	0.05	0.7
	Real_BP	3.5*	0.001	0.8
	Control	4.9*	<0.001	1.5
VE_ZSwing	VE_BP	3.6*	0.001	1.1
	Real_BP	2.7	0.009	0.8
	Control	5.3*	<0.001	1.7
VE_OSwing	VE_BP	–2.4	0.019	0.7
	Real_BP	–1.8	0.09	0.5
	Control	–4.4*	<0.001	1.4
Real_Hits	VE_BP	3.6*	0.001	1.0
	Real_BP	3.4*	0.001	0.7
	Control	6.1*	<0.001	1.2
Real_ZSwing	VE_BP	3.2*	0.002	1.0
	Real_BP	0.43	0.667	0.1
	Control	2.8	0.009	0.9
Real_OSwing	VE_BP	–3.7*	0.001	1.2
	Real_BP	–3.1*	0.005	0.8
	Control	–5.5*	<0.001	1.7
PR_Type	VE_BP	3.6*	0.001	1.4
	Real_BP	3.1*	0.005	1.1
	Control	4.3*	<0.001	1.6
PR_Strike	VE_BP	–1.9	0.056	0.6
	Real_BP	0.80	0.43	0.3
	Control	2.7	0.01	0.8

*^∗^Significant at corrected *p* = 0.006.*

#### Retention

To evaluate the degree to which post-training performance was retained, post-test and retention scores were compared for dependent variables using pairwise *t*-tests with Bonferroni correction (critical *p* = 0.008). This analysis revealed no significant differences for any of the training groups, *p*s all > 0.05, *d*s all < 0.5.

### Five-Year Follow Up

**FIGURE 5:**
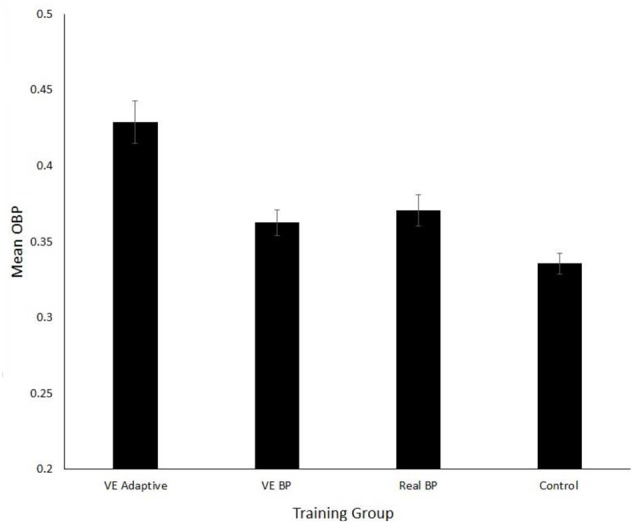
Mean on-base percentage (OBP) for the four training groups. Error bars are standard errors.

**Figure 5** shows the mean season OBP for each player’s high school season following the training. For these data, two participants (one from the VE batting practice and one from the control group) were removed because they played in fewer than five games due to injury. The one-way ANOVA performed on these data revealed a significant main effect of training group, *F*(3,74) = 10.8, *p* < 0.001, $\eta_{p}^{2}$ = 0.30. Independent samples *t*-tests with Bonferroni correction (critical *p* = 0.017) revealed that OBP was significantly higher for the VE adaptive as compared to the VE batting practice, *t*(37) = 3.7, *p* = 0.001, *d* = 1.2, and the control group, *t*(37) = 4.8, *p*< 0.001, *d* = 1.8. There was a marginally significant difference (with a medium-large effect size) between the VE adaptive and real batting practice group, *t*(38) = 2.5, *p* = 0.025, *d* = 0.7.

The multiple linear regression performed on OBP indicated that four predictors explained 68% of the variance (*R*^2^ = 0.47, *F*(8,69) = 9.7, *p* < 0.001). The significant predictors were ΔVE hits (β = 0.013, *p* = 0.01), Δreal hits (β = 0.0.07, *p* = 0.045), Δreal O-Swing % (β = -0.08, *p* = 0.041), and Δpitch type accuracy (β = 0.02, *p* = 0.021). ΔVE hits explained the highest amount of variance followed by Δpitch type accuracy.

The number of participants that played at least one full season at a level higher than high school baseball in the 5 years following the study was as follows: VE adaptive, 8 (1 AA, 1 A, 4 NCAA, 2 NCJAA); VE batting practice, 1(NCAA); Real batting practice, 3(1 A, 1 NCAA, 1 NCJAA); and Control, 1 (NCAA). A Chi-square test of proportions revealed that this distribution is significantly different from equality, χ^2^ = 7.9, *p* = 0.047.

## Discussion

The goal of the present study was to evaluate the transfer of perceptual-motor skills trained in an adaptive baseball batting VE to real baseball performance. The VE adaptive training used in the present study was superior to the other types of training investigated in many ways. Players in the VE adaptive group showed significant improvements for 7/8 of the batting performance assessments and these improvements were maintained in a 1-month retention test. Consistent with hypothesis (i), for the majority of the assessments, the magnitude of improvement was significantly greater than what was found for the other three groups in the study. Finally, consistent with hypotheses (ii) and (iii), the batting performance in the full season following the training (as assessed by OBP) was significantly higher for VE adaptive group as compared to the other groups and a significantly higher proportion of players in the VE adaptive group reached levels of competition above high school. Therefore, the results of the present study provide evidence for both near and far transfer of training for a baseball batting VE.

In looking at the level of play reached by the participants in the study it is interesting to compare to general trends for US high school players ^3^. It has been estimated that about 6% of high school players go on to play NCAA college baseball and only about 0.5% are drafted by a MLB team. Comparable numbers for players in the VE adaptive training group of the present study were 20 and 10%. Although these values cannot be compared statistically, this result does again suggest good transfer of training to real baseball.

When examining transfer of training effects it is important to consider the underlying perceptual-motor and cognitive mechanisms. This can be done in a few different ways using the data from the present study. First, consider the differences in results between the VE adaptive and VE batting practice groups. The significant differences in the magnitude of performance improvements, OBP, and the results of the 5-year follow up suggest that the benefits of the VE adaptive training in the present study were not simply due to the fact that it provided more repetition of hitting practice relative to the control group. Looking more closely, both types of VE training lead to increases in the number of hits in the VE test that were not statistically different in magnitude. However, there were two primary differences between these groups. First, as shown in **Figure 4**, the VE adaptive training resulted in significant improvements in performance on the real batting tests while the VE batting practice training did not. In other words, the results of the present study suggest that simply performing multiple hitting repeats in a VE has poor transfer to real batting. Second, batters in the VE Adaptive group exhibited greater knowledge of the strike zone (as shown by a greater increase in Z-Swing %) and superior pitch type recognition as compared to the VE batting practice group.

For the VE adaptive and real batting practice training, the group differences were smaller than what was found for the two VE training groups. Similar to the VE adaptive training, real batting practice lead to significant improvements in 7/8 of the batting performance assessments. Notably, real batting practice lead to significant changes in the number of hits and O-Swing % in the VE batting tests. Therefore, there was an asymmetry in the results of the present study with real batting practice leading to improvements in some aspects of virtual batting but not vice versa. Turning to a comparison of the magnitude of improvements from pre–post training, not surprisingly VE adaptive training did result in significantly more hits in the VE batting test than real batting training. But it also resulted in significantly greater improvements in the number of hits and O-Swing % in the real batting test, and significantly better pitch-type recognition.

A final way of examining the perceptual-motor mechanisms underlying the transfer effects found in the present study is via the multiple regression analysis quantifying the relationship between OBP and the performance assessment variables. When the data for all groups were used, there were two types of significant effects that were observed. First, perhaps not surprisingly, batters that showed the greatest improvements in the number of hits achieved (both in the VE and real tests) had better batting performance in league play. Taken on its own this effect could be explained in multiple different ways. For example, perhaps those participants that showed the greatest improvements on the hitting tests were also those players that put more effort into the regular team practice or were more motivated.

The second type of effect seen in the multiple regression analysis suggests there were also some improvements related to the mechanisms underlying batting skill, however. Specifically, improvements in pitch-type recognition and O-Swing % as a result of training were also significantly related to OBP. The change in O-Swing % (i.e., the likelihood the batter swings at a pitch that is outside of the strike zone) is particularly notable because it was not directly targeted in the tests or training, e.g., batters were not explicitly told that they should swing only at strikes. Instead, this improvement seemed to be a positive side effect of the training manipulations. These findings are consistent with recent research that has shown significant correlations between batting performance, plate discipline, and pitch recognition in professional baseball batters (Morris-Binelli et al., in press).

Taken together, the results of the present study suggest that the VE adaptive training lead to some key perceptual-motor changes which underlie the improvements. First, the changes in pitch recognition ability, O-Swing %, and Z-Swing % described above all suggest that the training resulted in greater sensitivity to visual information provided by the ball in flight. Specifically, these findings suggest that the VE adaptive training resulted in an improved ability to use the pattern of lace rotation to recognize the pitch type (Hyllegard, 1991; Gray, 2002) and an improved ability to use monocular cues to direction of motion in depth to determine whether or not a pitch would cross the plate in the strike zone (Gray, 2002). It is further proposed that these improvements were facilitated by the use of the staircase procedure in training. Overall, batters in the VE adaptive group were exposed to combinations of pitch types and trajectories that are more representative of the range of conditions they face in game play. However, rather than facing the full range of conditions right away, the staircase procedure presumably facilitated better learning by keeping challenge at an appropriate level (Guadagnoli and Lee, 2004). Finally, it is possible that the conditions in the VE adaptive training promoted a greater degree of exploration of the perceptual-motor space leading to a better calibration between the motor responses involved in producing a swing to a particular location and the visual information about the ball flight (Davids et al., 2008).

There are some important limitations of the present study that will need to be addressed in future research. First, the definition of a successful “hit” in present study (homerun or ball that travels to the outfield) does, of course, not match with what is used in games. The choice to use this definition was primarily a practical one (i.e., the difficulty of recruiting/simulating the other seven fielders). For the real batting test, this would have also added a further complication in that the performance on the batting tests would depend partially on the skill of the fielders. A more effective solution in future research might be to use ball tracking technology to calculate quality of contact variables (e.g., launch angle and exit velocity) as metrics of hitting performance.

A second limitation is that the present study did not include a real batting training group in which challenge and variability were manipulated in a similar manner to what was done with the VE adaptive group. On a theoretical level, it would be interesting to determine if these practice principles have similar effects in real and virtual training. However, this condition was not included in the present study because, while not impossible to recreate on the field, using the staircase procedure and randomly interleaving pitch types is highly impractical and is, therefore, very unlikely to be adopted in real practice.

A third limitation was the variation of pitcher “handedness” in the real batting training and testing. Obviously, there are more differences between a left-handed vs. right-handed pitcher than the horizontal release point varied by moving a pitching machine. Therefore, it is possible that one of the reasons for the difference in results for the VE adaptive and real batting practice groups was that the former group received opportunities to view the (simulated) delivery of a left-handed pitcher in training while the latter group did not. However, it is argued that this effect cannot explain all of the differences between these groups or the VE batting practice group (which also faced the simulated left-handed pitcher) would have also had superior results to the real batting practice group. As can be seen in **Figures 2–4**, this was clearly not the case.

A final point to consider is the fidelity of the simulation used in the present study. The physical fidelity of the VE used to collect the data presented here was clearly much lower than VR technology currently in use (e.g., CAVE and HMD systems). Specifically, the field of view was considerably smaller, the bat was not simulated, binocular information about ball flight was not included, and the flight of the ball was not simulated all the way to the plate. Although it was not measured, it is also likely that the degree of immersion was considerably lower. This raises the possibility that different (even greater) training benefits might be found using VE training systems that have a higher degree of physical fidelity and immersion. However, to date, there is little if any evidence to support the assumption that higher physical fidelity and greater immersion leads to more effective training (reviewed in Gray, in press). Furthermore, along with the present results, effective transfer of training has been found for relative low fidelity simulations (e.g., Todorov et al., 1997; Lammfromm and Gopher, 2011). Clearly, more research is needed to determine the specific characteristics of sports training VEs that are important for achieving effective transfer of training.

The present study adds to the (slowly) growing body of evidence on the effectiveness of VE training for sports (reviewed in Gray, in press). It provides evidence of positive, near (performance on tests similar to the training procedures), and far (performance in league play and competition level reached) transfer of training. As has been discussed in the context of research (e.g., Zaal and Bootsma, 2011), the present findings also suggest that the real value of using VE as a training tool for sports is not the ability to create more repetitions of the same types of practice that area used in real training. Instead, the real return on investment for developing a sports VE is likely to come from the ability to create unique, evidence-based training conditions that are impossible or highly impractical to use in real training.

## Author Contributions

The author confirms being the sole contributor of this work and approved it for publication.

## Conflict of Interest Statement

The author declares that the research was conducted in the absence of any commercial or financial relationships that could be construed as a potential conflict of interest.

## Footnotes

1 A VE is defined as a system which includes the following elements: (i) a projection/display system which can present an image with a relatively large field view (FOV) such as a Head Mounted Display (HMD) or a Cave Automatic Virtual Environment (CAVE), (ii) some means of tracking the user’s movements to allow for interaction with objects in the virtual world such as a motion capture system that tracks markers placed on the user’s body, a cyber glove which tracks hand movements, or a motion-tracked effector like a bat or racquet, (iii) some means for providing feedback to the user about their actions such visual (e.g., ball flying on the field), auditory (e.g., sound of bat-ball contact) and/or haptic (e.g., vibration of force feedback from contact), and (iv) software used to render a 3D computer generated model of a sports-specific environment. Research on transfer for sports training systems which include some but not all of this elements, although potentially still valuable, is not considered here. Examples include video occlusion training of anticipation/decision-making skills (e.g., Abernethy, 1991) which is typically non-interactive due to the use of video and the Neurotracker system (Romeas et al., 2015) which involves passive user responses, is not interactive, and does not use sports-specific stimuli. For more discussion of this issue please see Gray (in press).

2 Note, Z-Swing which is used to indicate a pitch inside the strike “Z”one and O-Swing which is used to indicate a pitch “O”utside the strike are used here to be consistent with the terminology currently used in baseball analytics (https://www.fangraphs.com/library/offense/plate-discipline/).

3 http://www.hsbaseballweb.com/probability.htm
